# Unraveling biotypes of the northern house mosquito, *Culex pipiens* s.l. (Diptera: Culicidae): molecular differentiation and morphometric analysis

**DOI:** 10.1093/jisesa/ieae006

**Published:** 2024-02-10

**Authors:** Ingrid E Alvial, Raquel Hernández-P, Manuel J Suazo, Christian R González, David Véliz, Hugo A Benítez

**Affiliations:** Laboratorio de Ecología y Morfometría Evolutiva, Centro de Investigación de Estudios Avanzados del Maule, Universidad Católica del Maule, Talca, Chile; Departamento de Ecología Evolutiva, Instituto de Ecología, Universidad Nacional Autónoma de México, Ciudad de México, México; Instituto de Alta Investigación, Universidad de Tarapacá, Arica, Chile; Instituto de Entomología, Facultad de Ciencias Básicas, Universidad Metropolitana de Ciencias de la Educación, Santiago, Chile; Departamento de Ciencias Ecológicas, Universidad de Chile, Ñuñoa, Santiago, Chile; Centro de Ecología y Manejo de Islas Oceánicas (ESMOI), Coquimbo, Chile; Laboratorio de Ecología y Morfometría Evolutiva, Centro de Investigación de Estudios Avanzados del Maule, Universidad Católica del Maule, Talca, Chile; Centro de Investigación en Recursos Naturales y Sustentabilidad (CIRENYS), Universidad Bernardo O’Higgins, Santiago, Chile

**Keywords:** biotype, wing shape, geometric morphometrics, northern house mosquito

## Abstract

Geometric morphometrics was used to determine whether geographic isolation could explain differences in wing size and shape between and within continental (27°S to 41°S) and insular (Rapa Nui) populations of *Culex pipiens* s.s. Linnaeus and their biotypes (f. *pipiens* and f. *molestus*). Molecular protocols based on polymorphisms in the second intron of nuclear locus ace-2 (acetylcholinesterase-2) were used to differentiate *Cx. pipiens* s.s. from *Cx. quinquefasciatus* Say, and an assay based on polymorphisms in the flanking region of a microsatellite locus (CQ11) was used to identify biotypes. *Culex pipiens* f. *molestus* and hybrids shared larval habitats in all continental sites, while *Cx. pipiens* f. *pipiens* was found in 5 of the 10 sites. Only biotype *molestus* was found in Rapa Nui (Easter Island) *Pipiens* and *molestus* biotypes occur sympatrically in aboveground locations, and only *molestus* was found in the underground site (ME). Biotype *molestus* was dominant in rural locations and preferably anthropophilic. These results agree with the ecological descriptions previously reported for the biotypes of *Cx. pipiens* s.s. Procrustes ANOVA only showed differences in centroid size between biotypes in females and males and did not show significant differences in wing shape. However, we found significant differences among the geographic areas in the centroid size and wing shape of both females and males. Particularly, the population of Rapa Nui Island had shorter wings than the continental populations. The results highlight the effects of geographic and environmental processes on morphotypes in vector mosquitoes.

## Introduction

Mosquitoes of the genus *Culex* are important vectors of human diseases. *Culex pipiens* Linnaeus and *Cx. quinquefasciatus* Say are considered the primary vectors of the West Nile virus (WNV), a flavivirus introduced in the United States in the 1990s (Fonseca et al. 2004). WNV spread to Central America and the Caribbean between 2001 and 2004. In South America, WNV has been reported in horses in Argentina (Morales et al. 2006) and flamingos in Colombia (Osorio et al. 2012), and there is strong evidence of ongoing WNV transmission throughout South America, including Brazil and Venezuela (Lorenz and Chiaravatolli 2022).

*Culex pipiens* and *Cx. quinquefasciatus* are both species of the *Cx. pipiens* complex and are found in South America. They are highly similar morphologically but can be distinguished by the shape of the male terminalia (Dobrotworsky 1967); however, the lack of physical morphological differences in females, coupled with the existence of hybrids make identification challenging (Price and Fonseca 2015). Molecular studies using mitochondrial markers have not been successful in distinguishing between members of this complex (Laurito et al. 2013). Specific protocols based on a nuclear locus developed by Smith and Fonseca (2004) allowed the differentiation of *Cx. pipiens* s.s. from *Cx. quinquefasciatus*. Polymorphisms of the CQ11 microsatellite locus (a repeated TG sequence) have been used to differentiate between 2 biotypes or ecological forms of *Cx. pipiens*: f. *pipiens* and f. *molestus* (Bahnck and Fonseca 2006). Studies of *Cx. pipiens* biotypes have revealed behavioral and physiological differences that strongly influence their vectorial capacity (Ciota and Kramer 2013). These differences include preferences in larval habitat hypogeous (biotype *molestus*) vs. epigeous (biotype *pipiens*), rural (biotype *pipiens*) vs. urban (biotype *molestus*), and preferences in adult strategies, such as urban variations in host feeding patterns (mammophilic vs. ornithophilic), gonotrophic development (autogeny vs. anautogeny), and means or presence of adult female hibernation (quiescence vs. diapause) (Farajollahi et al. 2011). *Culex pipiens* f. *pipiens* was described mainly as anautogenic, ornithophilic, heterodynamic, and eurygamous, while *Cx. pipiens* f. *molestus* has been described as autogenic, mammophilic, homodynamic, and stenogamous (Shaikevich et al. 2016, Aardema et al. 2020).

Most studies recognize these biotypes as valid entities to infer patterns of diversity and divergence (Shaikevich et al. 2016, Vogels et al. 2016, Haba and Macbride 2022), but their validity and taxonomic position remains controversial. Given the difficulty and cost of molecular analysis, and the debate surrounding the use and validity of biotypes, geometric morphometric (GM) methods have emerged as robust tools for differentiating cryptic species within the *Cx. pipiens* complex (Wilke et al. 2016, Simões et al. 2020). Studies of GM differentiate epidemiologically relevant mosquitoes, particularly by comparing wing shape, as vein intersections can be used as conspicuous landmarks, and are homologous between species (Lorenz et al. 2017). Wing-shaped studies have discerned taxonomic issues in the family Culicidae as differences within (Motoki et al. 2012, Gómez et al. 2014) and between species (Laurito et al. 2015, Wilke et al. 2016, Garzón et al. 2020). GM has also allowed differentiation of species in sympatric areas (Lorenz et al. 2012). Garzón et al. (2020) correctly separated females of *Cx. pipiens* s.s. and *Cx quinquefasciatus* from Argentina using 17 landmarks and the siphonal index. These authors described that *Culex quinquefasciatus* had thinner wings than *Cx. pipiens*, which may indicate ecological adaptations to the environment in its distribution. Wu et al. (2014) found that *Cx. pipiens* f*. molestus* could be distinguished from *Cx. quinquefasciatus* and intermediates by characters of the female wing (intersection of the subcostal vein with the costal in relation to the level of furcation of R1 + 2), abdominal terga, male scutum, and phallosomes. Krtinic et al. (2015) found significant differences in the number of pecten spines and siphonal index between biotypes (*pipiens* and *molestus*) for both sexes. Thus, morphological differentiation based on wing shape aids in taxonomic analyses of biotypes in the *Cx. pipiens* complex, especially in areas with potential for WNV expansion in South America (Figueroa et al. 2020).

This study aims to compare continental (from 27° S to 41° S latitude) and insular populations of *Cx. pipiens* s.s. using GM methods. Specifically, we compare wing size and wing shape and use these traits to evaluate differences between and within biotypes. Geographic isolation is an important factor in driving differences in wing shape among *Aedes albifasciatus* Macquart populations from the 3 ecoregions of Argentina (Garzón and Schweigmann 2018); thus, geographic isolation could determine differences in wing shape between the *Cx. pipiens* s.s. and its biotypes along continental and insular sites in South America.

## Materials and Methods

### Collection and Processing

Larvae of *Cx. pipiens* s.s. were collected from artificial habitats, mainly urban and suburban cemeteries, located between 27° S and 41° S, and from seminatural ponds (Table 1). Cemetery vases were monitored until reaching at least 100 individuals per locality (about 3–6 vases). Adults were collected from underground and aboveground habitats in rural areas using a hand-held vacuum cleaner. All larvae collected were transported in containers with water from the site where they were collected. Then larvae were transferred to 300 cc plastic containers (randomly 50 larvae per container) prepared with demineralized water and fish food was added daily. Emergent adults were transferred using a mouth aspirator, killed in a deep freezer, and identified. *Culex pipiens* complex specimens were identified under a stereomicroscope using the identification keys of Rossi and Almirón (2004), and González et al. (2015, 2016).

**Table 1.** Collection of *Cx. pipiens* s.s. mosquitoes identified and used for molecular and morphometric analysis. Sites are grouped by geographic area (N: north, C: central, S: south, and I: island) as indicated in brackets

Sample ID	Site	Geographic coordinates	Climate area	Altitude meters	Sampling date	Sample type and habitat	Development status	*N* total	Sample size
TB (N)	Tiltil bajo, Copiapó	27°22ʹ43″ S 70°18ʹ13″ W	Desert	415 m	26-11-2021 (late spring)	Seminatural ponds with vegetation scrub in urban environment	Larvae	295	18
CC (N)	Cementerio Municipal de Copiapó	27°22ʹ18″ S 70°20ʹ16″ W	Desert	383 m	29-11-2021 (late spring)	Vases without/with remains of flowers in partial shade in urban cemetery	Larvae	62	18
CS (N)	Cementerio Parque La Foresta, La Serena	29°55ʹ26″ S 71°11ʹ16″ W	Semi-desert coast	149 m	08-12-2021 (summer)	Vases with remains of flowers in partial shade in suburban cemetery	Larvae	245	20
MC (N)	Cementerio Municipal de Coquimbo	29°58ʹ03″ S 71°19ʹ50″ W	Semi-desert coast	14 m	09-12-2021 (summer)	Vases with remains of flowers in partial shade in urban cemetery	Larvae	268	20
PA (C)	Cementerio de Playa Ancha	33°01ʹ39″ S 71°38ʹ45″ W	Mediterranean coast	83 m	12-03-2022 (late summer)	Vases with remains of flowers without shade in urban cemetery	Larvae	32	13
SA (C)	Cementerio Bajos de Mena Santiago	33°36ʹ50″ S 70°36ʹ09″ W	Mediterranean valley	661 m	17-11-2021 (late spring)	Vase with remains of flowers in partial shade in urban cemetery	Larvae	189	20
ME (C)	Los Maitenes, Melipilla	33°49ʹ49″ S 71°23ʹ11″ W	Mediterranean valley	140 m	19-12-2021 (summer)	Adults in sewer subway in rural environment	Adults	300	20
TH (S)	Trehuaco, Ñuble	36°22ʹ50″ S 72°48ʹ41″ W	Mediterranean valley	18 m	15-07-2021 (larvae) and 25-01-2022 (adults)	Adults inside rural home and larvae in artificial container	Larvae and adults	124	20
PM (S)	Cementerio Parque Esperanza, Puerto Montt	41°28ʹ26″ S 72°55ʹ41″ W	Rainy coast	29 m	24-01-2022 (summer)	Vase with remains of flowers in partial shade in urban cemetery	Larvae	53	20
RN (I)	Hanga Roa, Rapa Nui	27°09ʹ02″ S 109°25ʹ55″ W	Tropical island	41 m	10-09-2022 (adults) and 18-10-2022 (larvae)	Adults in semirural home and larvae in plastic containers	Larvae and adults	26	25

### Molecular Analysis

Once the sex of each specimen was determined and the wings removed, the whole body was used to extract DNA with the method described by Aljanabi and Martinez (1997). The protocols of Smith and Fonseca (2004), and Bahnck and Fonseca (2006) were used to distinguish *Cx. pipiens* s.s. from *Cx. quinquefasciatus* and to identify biotypes and hybrids within *Cx. pipiens* s.s., respectively.

To confirm *Cx. pipiens* s.s., we used primers (ACEpipF, ACEquinF, and B1246S) and the PCR protocol described by Smith and Fonseca (2004). PCR was performed in a 20 μl volume using 0.12 μl Taq, 0.5 μl BSA, 0.5 μl MgCl_2_, 2.4 μl dNTP, 1.3 μl Buffer, 0.5 μl primer ACE pip for *Cx. pipiens*, 0.75 μl primer ACE quin for *Cx. quinquefasciatus*, and 0.75 μl primer B1246s, 2 μl genomic DNA, and 11.2 μl pure water (Milli-Q). The PCR was performed with an initial denaturation at 94 °C for 5 min, followed by 35 cycles at 94 °C for 30 s, 55 °C for 30 s, and 72 °C for 1 min, with a final 5 min extension at 72 °C. Amplified fragments were visualized on 1% agarose gels: a single DNA fragment of 600 bp corresponds to *Cx. pipiens* s.s. a single DNA fragment of 300 bp corresponds to *Cx. quinquefasciatus*. Individuals showing both fragments are considered as hybrids between species. To confirm *Cx. pipiens,* we sequenced the cytochrome oxidase I gene (COI) using the primers C1-N-2191 (5ʹ-CCG GTA AAA TTA AAA TAT AAA CTT C-3ʹ) and C1-J-1718 (5ʹ-GGA GGA TTT GGA AAT TGA TTA GTT CC-3ʹ) described for insects by Simon et al. (1994). Our PCR protocol contained 2 µl DNA (50 ng/µl), 2.5 µl 10XPCR buffer (Invitrogen), 1.6 µl MgCl_2_ (50 mM) (Invitrogen), 2 µl dNTPs (2.5 mM) (Invitrogen), 14.6 µl water, 1 µl forward and reverse primers (50 ng/µl), and 0.3 µl Taq DNA polymerase (Invitrogen). The procedure started at 95 °C for 2 min, followed by 35 cycles of 95 °C for 30 s, 50 °C for 30 s, 72 °C for 30 s, and a final step of 72 °C for 5 min. Amplifications were verified on 1% agarose gels and sequencing in one direction was performed at the sequencing service of the Pontificia Universidad Católica de Chile. Sequences were compared with published sequences in GenBank.

Once *Cx. pipiens* s.s. was confirmed, the CQ11 locus was amplified to differentiate biotypes. The PCR were carried out according to Bahnck and Fonseca (2006), using the pipCQ11R, molCQ11R, and CQ11F primers. The PCR assay used was standardized for a 20-µl reaction volume. Reactions contained 0.12 μl Taq, 0.5 μl BSA, 0.5 μl MgCl_2_, 2.4 μl dNTP, 1.3 μl buffer, 0.5 μl primer pipCQ11R for biotype *pipiens*, 0.75 μl primer molCQ11R for biotype *molestus*, and 0.75 μl primer CQ11F, 2 μl genomic DNA, and 11.2 μl free water. The PCR was performed with an initial denaturation at 94 °C for 5 min, followed by 40 cycles at 94 °C for 30 s, 54 °C for 30 s, and 72 °C for 40 s, with a final extension at 72 °C for 5 min. Amplified fragments were visualized on 1% agarose gel: a single DNA fragment of 200 bp corresponds to the *pipiens* biotype, a single DNA fragment of 250 bp corresponds to the *molestus* biotype and individuals presenting both fragments were considered as hybrids.

### Geometric Morphometrics Analysis

**Fig. 1.**
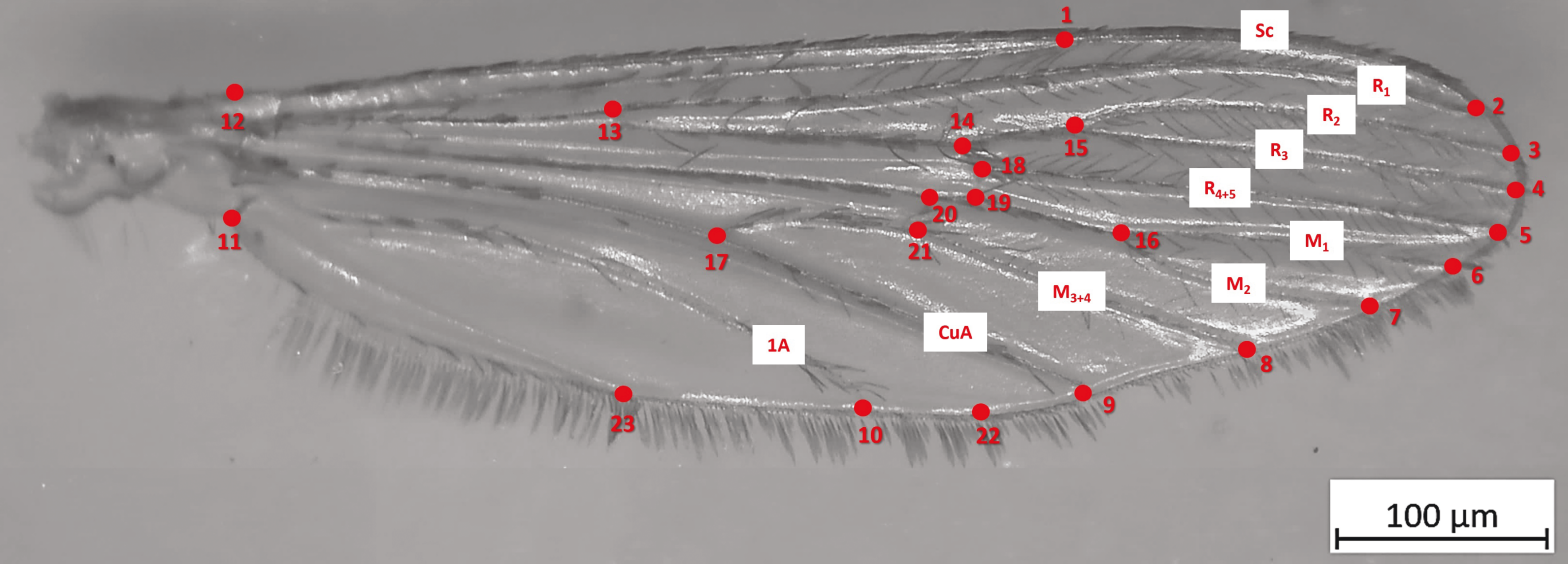
Twenty-three landmarks on the wing of an *Culex pipiens* s.l. mosquito for GM analyses.

The right wing of each specimen was removed and mounted a glass microscope slide with a 0.08- to 0.12-mm glass coverslip. *Culex* wings were then photographed using a Stemi 508 Zeiss digital camera connected to a stereomicroscope with the Axiocam program. A total of 23 landmarks were digitized on the wings using the software TPSUtil v1.58 and TPSDig v2.17 (Rohlf 2015). Landmarks along the main veins were chosen, so that the configurations were repeatable, appropriate for each wing, and to cover the wing shape as completely as possible (Fig. 1). The landmark selection was consistent with other studies analyzing mosquito wing morphometrics (Lorenz et al. 2017, Martinet et al. 2021).

Multivariate analyses were performed using MorphoJ v. 1.07a (Klingenberg 2011) and the R program (R Core Team 2016). The raw landmark coordinates for all individuals were aligned and superimposed in MorphoJ, using the Procrustes superimposition function to remove variation due to differences in scale, position, and orientation from the coordinates (Rohlf and Slice 1990). Centroid size is defined as the square root of the sum of the squared distances between the centroid and each landmark and can be used as a proxy for wing size (Bookstein 1997). So, the centroid size and Procrustes coordinates obtained from landmark data were used for further statistical analyses.

Wing size and shape were analyzed between biotypes (*molestus*, *pipiens*, and hybrids) and between geographic areas: northern area (27°S–29°S), central area (sites located around 33°S), southern area (36°S to 41°S), and island (Rapa Nui), using the same set of individuals. Environmental and climatic conditions are similar within each of these areas (Sánchez and Morales 2002).

To visualize the relative morphological changes between populations, a warped outline drawing was made in TPSDig2 (Rohlf 2015), which will be used to visualize the mean shape of *Culex* specimens. Then a covariance matrix of individuals was created in R using libraries geomorph, Morpho, plyr, dplyr, and ggplot2, to perform a principal component analysis (PCA) and an average PCA of each biotype and geographic area, and canonical variate analysis (CVA). CVA was performed through 10,000 permutations of Mahalanobis distance from the pooled within-location covariance matrix. The mean shape of *Cx. pipiens* s.s. and biotypes per geographic area were extracted, and the covariance matrix of mean shape variation was used to identify graphically morphological variation between geographic areas and biotypes. The allometric influence of wing size on wing shape was assessed by multivariate regression of the Procrustes coordinates against centroid size, using a permutation test with 10,000 randomizations (Monteiro 1999). A comparative analysis of the centroid sizes was performed for populations of both sexes independently using a Procrustes analysis of variance (ANOVA), with 1,000 permutations using *procD.lm* (geomorph function).

## Results

### Molecular Analysis

Locus ace-2 showed a 600 bp DNA fragment for all individuals; thus, 100% of samples were identified as *Cx. pipiens* s.s. Sequences of the COI gene obtained from some individuals were contrasted with these in GenBank; we confirmed *Cx. pipiens* in all individuals analyzed. The microsatellite CQ11 was successfully amplified in 153 of the 194 specimens studied. This locus showed the presence of both alleles (200 and 250 bp) and both homozygotes and the heterozygote in the samples. Of these, 58.8% were identified as *Cx. pipiens* f. *molestus*, 11.8% were *Cx. pipiens* f. *pipiens*, and 29.4% were classified as hybrids between the two.

**Fig. 2.**
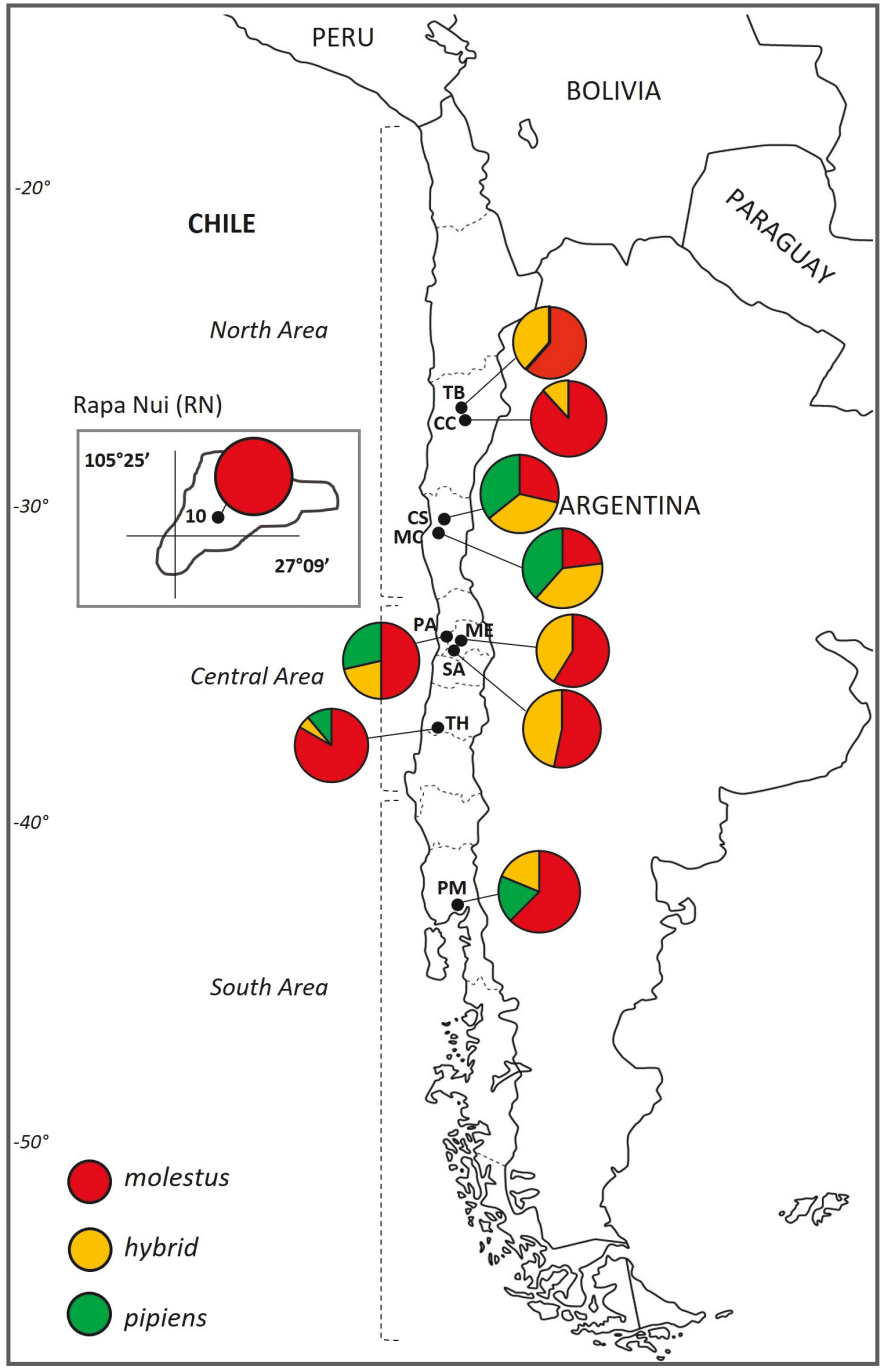
Composition of *Culex pipiens* genotypes from ten field-collected populations in Chile using the CQ11 microsatellite assay. The nine continental sites and one insular site (Rapa Nui) are indicated by blacks dots.

Biotype *molestus* was present in all collection sites, while biotype *pipiens* was present in 5 of the 10 localities (Fig. 2). Notably, biotype *pipiens* was observed in the coastal localities (<10 km from the coast). However, only biotype *molestus* was identified in Rapa Nui. Biotype *pipiens* was not found underground in Melipilla (ME) or the sampling sites of Copiapó (CC and TB). Adults from Trehuaco were all females collected after biting in a rural house; 2 of the 18 specimens were described as *Cx. pipiens* f. *pipiens.*

### Geometric Morphometrics Analyses

The proportion of shape variance that was explained by size (allometry) was found to be 4.44% and 7.65% in the male and female, respectively. This shows an effect of size on shape variance. ANOVA showed a significant wing shape effect related to sexual dimorphism (*F*_42_ = 93.19, *P* < 0.0001), which was not determined by allometry. Analysis was carried out separately females and males.

### Analysis by Biotypes

**Fig. 3.**
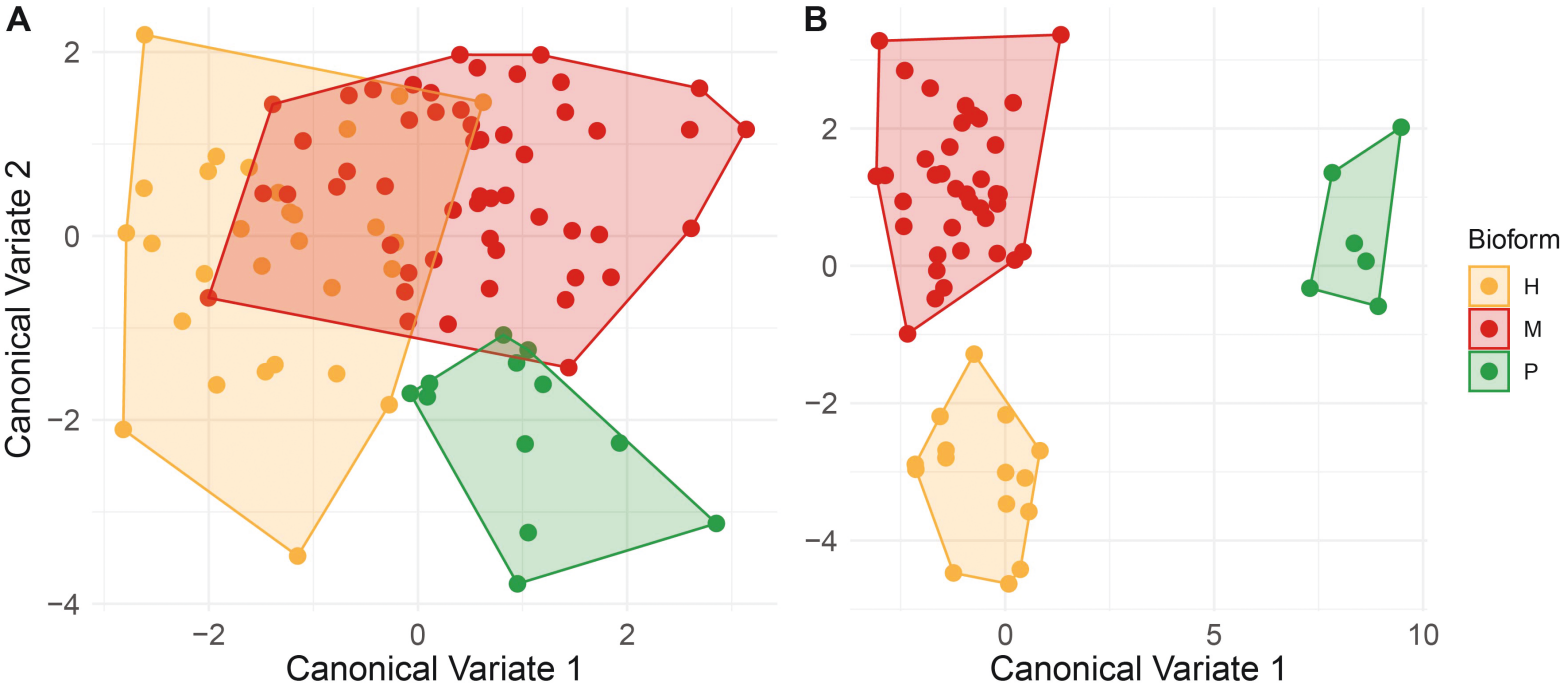
Canonical variate analysis of *Cx. pipiens* s.l. between biotypes to females (A) and males (B). Each colour represents a biotype (hybrid: yellow, *molestus*: red and *pipiens*: green).

**Fig. 4.**
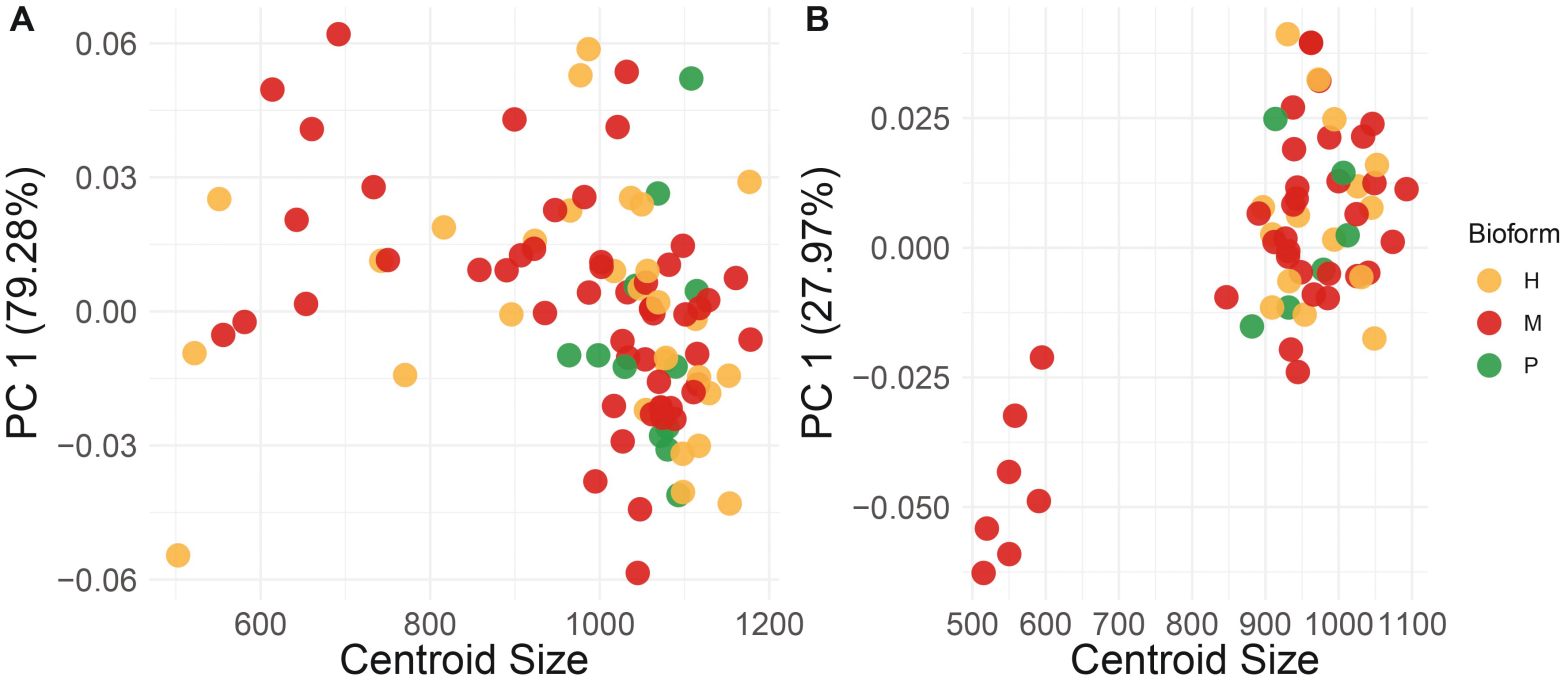
Shape variation accumulated in principal component 1 due to centroid size among biotypes of females (A) and males (B) of *Cx.pipiens* s.l.

In the CVA, the wing shape of females of *Cx. pipiens* s.s. differed among the 3 biotypes (H: hybrids, M: *molestus*, and P: *pipiens*) (Fig. 3A). The wing shape of the 3 biotypes in males was clearly segregated in the first 2 canonical axes, with more similarity between the wing shape of hybrids, and *molestus* individuals compared to *pipiens* (Fig. 3B). Procrustes ANOVA showed differences in the centroid size between biotypes in females (*F*_3_ = 3.15, *P* = 0.0276) and males (*F*_126_ = 2.15, *P* = 0.0184), but not in wing shape (Table 2). However, only individuals with biotype *molestus* from Rapa Nui showed shorter wings than the other biotypes, notorious in males (Fig. 4). The main differences between biotypes of the sexes of *Cx. pipiens* s.s. were associated with landmarks 1 (interception subcostal vein with costal vein) and 15 (furcation of R_2_ + 3).

**Table 2.** Procrustes ANOVA model of shape among biotypes for females (a) and males (b)

	SS	MS	df	*F*	*P*
(a) Females					
Centroid size					
Biotype	304,975.87	101,658.62	3	3.15	0.0276*
Individual	3,707,665.23	32,240.56	115		
Shape					
Biotype	0.012	0.000101	126	2.15	0.1339
Individual	0.227	0.000047	4,830		
(b) Males					
Centroid size					
Biotype	29.174	9.7248.70	3	3.56	0.0184*
Individual	1,910,808.14	27,297.25	70		
Shape					
Biotype	0.00877	0.000069	126	1.52	0.2504
Individual	0.13487	0.000045	2,940		

MS-mean sum of squares, SS-sum of squares, *significant *p* values.

### Analysis by Geographic Area

**Fig. 5.**
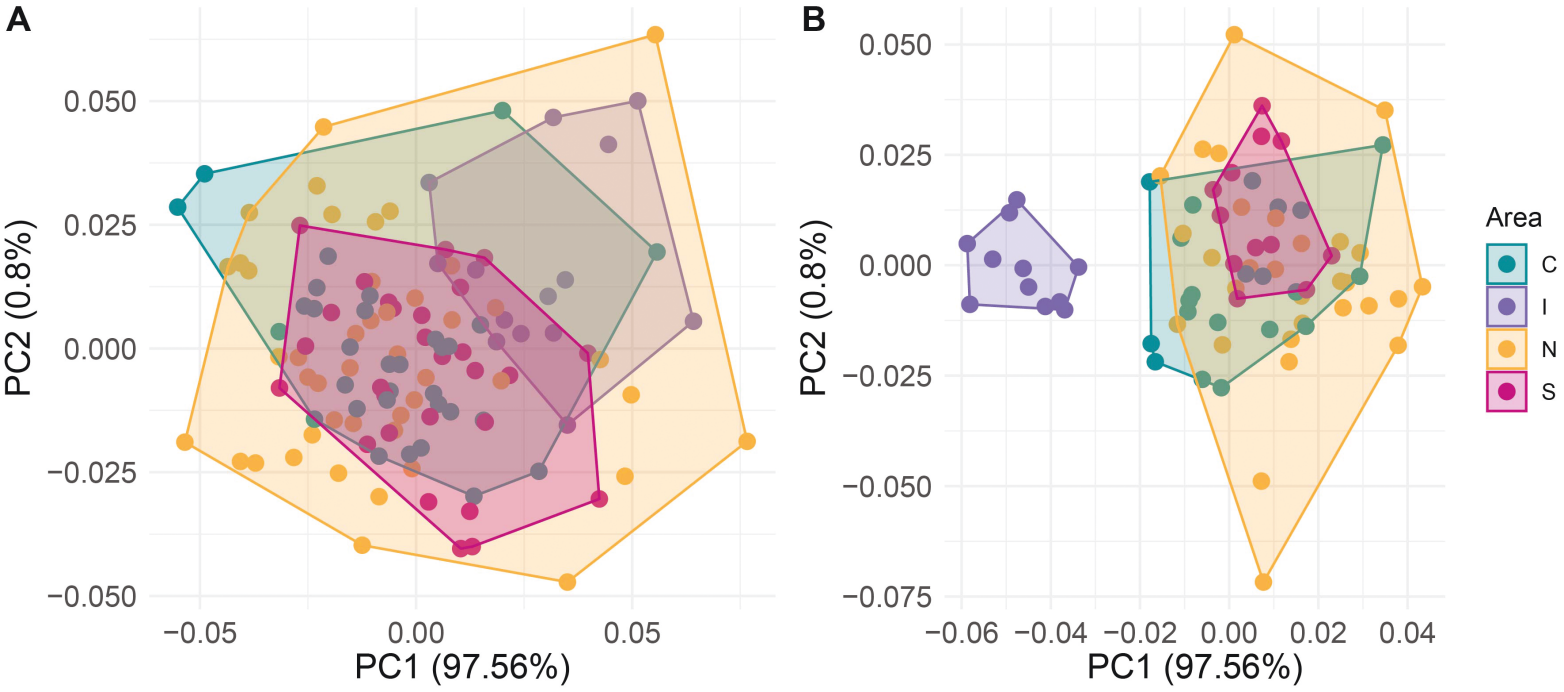
Results of Principal Component Analysis of females (A) and males (B). Each colour represents a geographical area (green: Central area, purple: Island, orange: North area and red: South area).

The first 2 components of the PCA accumulated 53.04% of the total variation of shape in females (PC1: 32.26% and PC2: 20.78%) (Fig. 5A) and 47.22% in males (PC1: 29.45% and PC2: 17.77%) (Fig. 5B). Male populations were clearly segregated, while female populations overlapped. Males from Rapa Nui showed a different wing shape than the mainland sites. In fact, males from Rapa Nui showed more compressed and smaller wings than those from the other sites, as indicated by the smaller distance between LM 16 and LM19. Procrustes ANOVA showed significant differences among geographic areas in centroid size and wing shape in both females and males (Table 3).

**Table 3.** Procrustes ANOVA model of shape variable among geographic area for females (a) and males (b)

	SS	MS	df	*F*	*P*
(a) Females					
Centroid size					
Area	1,564,406.549	5,214,68.849	3	24.49	<0.0001*
Individual	2,448,234.562	21,288.996	115		
Shape					
Area	0.02875	0.000228	126	5.22	<0.0001*
Individual	0.21109	0.000043	4,830		
(b) Males					
Centroid size					
Area	1,505,259.085	501,753.028	3	5.37	<0.0001*
Individual	697,295.182	9,961.359	70		
Shape					
Area	0.0348	0.000276	126	7.47	<0.0001*
Individual	0.1087	0.000037	2,940		

MS-mean sum of squares, SS-sum of squares, *significant *p* values.

The main relative changes between geographical areas were determined by LMs 1 (interception of subcostal vein with costal vein), 15 (furcation of R_2_ + R_3_), and 16 (furcation of M_1_ + M_2_). Major changes were observed in these landmarks in females and males. In the island sites, the subcostal intersected the costal before the furcation of R_2_ + R_3_, whereas at the other sites, the subcostal intersected the costal beyond the furcation of R_2_ + R_3_. Females and males of Rapa Nui have shorter wings than the continental individuals.

**Fig. 6.**
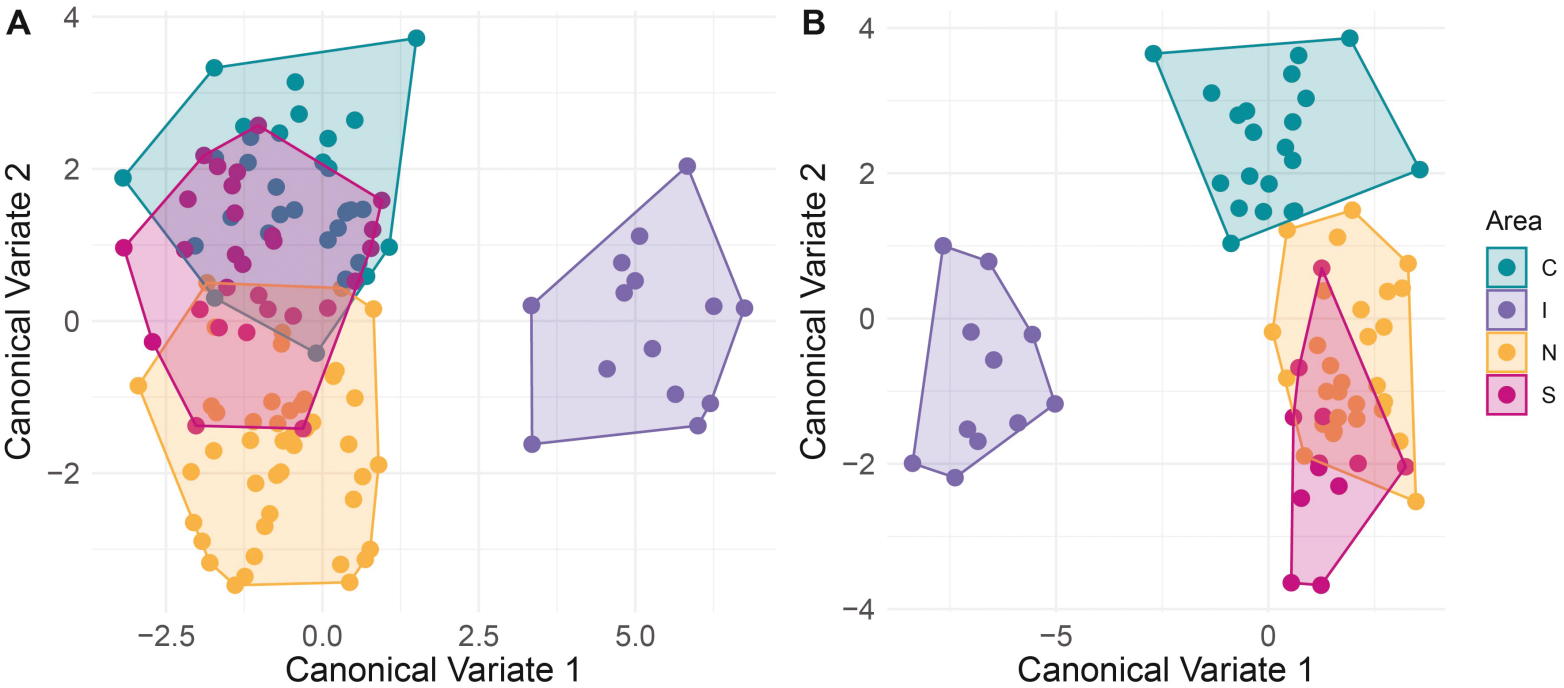
Canonical variate analysis of *Cx. pipiens* s.l. among geographical area (green: Central area, purple: Island, yellow: North area and red: South area) to females (A) and males (B).

The wing shape of females (Fig. 6A) and males in the CVA (Fig. 6B) differed among the 4 geographic areas (N: North, C: Central, S: South, and I: Island). The analysis showed a gradual differentiation among the continental sites in females, and all these sites differed in relation to the island. This pattern was also observed in the wings of males, and the shape of those from the island showed more differences compared to the other sites, followed by the individuals located in the central area.

**Fig. 7.**
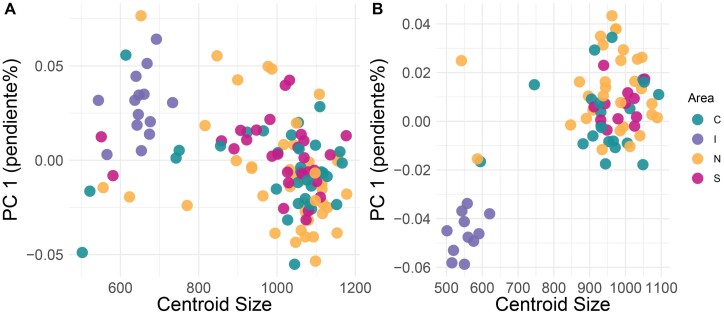
Shape variation accumulated in principal component 1 scores due to centroid size among geographical area (green: Central, purple: Island, yellow: North area and red: South area) of females (A) and males (B) of *Cx. pipiens* s.l.

The centroid size indicated that most mosquitoes from Rapa Nui had shorter wings than continental individuals (North, Central, and South populations), being more evident in males (Fig. 7).

## Discussion

The results of this study showed clear geographic variation in wing shape and morphology in *Cx. pipiens* s.s. Both biotypes and hybrids were recorded in all continental sites studied. The CQ11 assay amplified 78.8% of the specimens studied, identifying 58.8% of these as biotype *molestus*, 11.8% as biotype *pipiens*, and 29.4% as hybrids.

Both ecological forms of *Cx. pipiens* s.s. were found in sampling sites sharing the larval habitat and in both rural and urban habitats. The biotype *molestus* was found in an underground site and in aboveground sites, especially in a seminatural pond in Copiapó (TB). However, biotype *pipiens* was found only in aboveground sites, especially cemetery vases. This agrees with Vogels et al. (2016), who found *Cx. pipiens* f. *pipiens* and *Cx. pipiens* f. *molestus* in sympatry in aboveground locations.

Females from the Trehuaco site were collected after biting humans in a rural home, and 2 of the 18 specimens were described as *Cx. pipiens* f. *pipiens*. In the Northern Hemisphere, females of biotype *pipiens* primarily feed on birds and are therefore important vectors of pathogens circulating in bird populations to humans (Haba and Mcbride 2022).

Interestingly, relatively few larval habitats with biotype *pipiens* were identified, which could be associated with the number of samples in each area. On Rapa Nui, about 3,600 km west of the Chilean coast, all individuals were classified as biotype *molestus*. From its origin in the Mediterranean (according to the best supported hypothesis) *Cx. pipiens* f. *molestus* have spread significantly through human intervention, establishing worldwide distribution (Aardema et al. 2022) and reaching remote islands such as Rapa Nui. The first records of *Cx. pipiens* s.s. in Rapa Nui Island date back to the early 1900s (Fuentes 1914); its introduction was probably due to human transport from the American continent, as with the dengue vector *Aedes aegypti* Linnaeus, another mosquito present in Rapa Nui.

*Culex pipiens* s.s. and related species are probably the best studied examples of hybridization in mosquitoes. Hybridization is an intrinsically ecological process that can significantly influence different traits of individuals, such as response to abiotic environmental conditions, and as a biotic interaction, hybridization contributes to shaping patterns of biodiversity, and its spatial structure in species and communities (Porretta and Canestrelli 2023). Actual hybridization between mosquito species has been widely observed and elegantly demonstrated genetically (Savage and Kothera 2012). We found hybrids at all continental sites, mainly sharing larval habitats with the biotype *molestus*.

Diapause in *Cx. pipiens* s.s. has been well described (Robich and Denlinger 2005, Meuti et al. 2015). In the continental sites analyzed in this present study, *Cx. pipiens* f. *pipiens* was observed under different climatic conditions (summer and winter), ranging from an arid climate in the north to a temperate humid climate in the south. Therefore, specific studies of the diapause process will be necessary to understand the physiological requirements of *Cx. pipiens* s.s. in this part of its distribution.

Morphometric analysis of the biotypes showed significant differences only in wing centroid size for both females and males. Although we did not find significant differences in wing shape, it was possible to infer that some individuals of biotype *molestus* had shorter wings than biotype *pipiens* and hybrids, but these individuals were from Rapa Nui. Therefore, the differences observed in biotypes are associated with geographic area, and consequently, geographic isolation could be the principal driver that determines wing size in *Cx. pipiens* s.s. in South America.

To our knowledge, Krtinic et al. (2015) performed the first analysis assessing differences in wing shape between biotypes, describing differences in wing centroid size between females and males. A pairwise comparison showed that the wing centroid size of the hypogeous biotype was significantly smaller for both sexes compared to the epigeous biotypes. Other morphological characters, such as the siphonal index, have been commonly used to determine the taxonomic status of *Cx. pipiens* s.s. biotypes (Vinogradova 2003, Krtinić et al. 2015). However, an integrative approach including massive sequencing is necessary to clarify the taxonomic determination of the *Cx. pipiens* complex.

Procrustes ANOVA showed significant differences in centroid size and wing shape between the geographic areas studied. Individuals from the central and northern areas differed more, and all these locations differed from the island. In particular, the Rapa Nui population had shorter wings than continental populations.

The wing size and shape of insects are largely influenced by environmental factors such as temperature, relative humidity, larval density, and food availability (Morales-Vargas et al. 2010, Krtinić et al. 2015, Dellicour et al. 2017). However, since its introduction to the island, *Cx. pipiens* s.s. appears to have phenotypic variation related to geographic isolation, as described in *Pantala flavescens* Fabricius, another cosmopolitan insect present on the island (Alvial et al. 2019). The smaller wings observed in the insular population of this mosquito can be associated with the “island flightlessness hypothesis,” which is explained if a new species colonizing an isolated island is subject to a strong selective force against dispersal because flight could be disadvantageous in that the energy used to maintain and use the flight muscles is effectively wasted (Whittaker and Fernandez-Palacios 2007). Smaller wings might also indicate a reduction in body size (“island rule theory”), where body size is selected toward an optimal body size at which organisms maximize their reproductive output (Lokatis and Jeschke 2018). However, future studies on these topics are needed to confirm that this occurrence is greater than expected by chance alone.

In all comparative analyses, we found that the male populations were more spatially structured than the female populations. This pattern of sexual dimorphism has been previously recognized in culicid species (Virginio et al. 2015). Male mosquitoes do not require blood for reproduction or survival; they are completely dependent on sugars and the reserves they have acquired from larval feeding, in addition to adult food supply (Wilkerson et al. 2021). Therefore, food availability is critical for larval development. Some species produce smaller adults when food is limited (Carvajal-Lago et al. 2021). However, *Cx. pipiens* s.s. individuals were found in a microenvironment with an abundance of nutrients, so survival would not be dependent on nutrient availability. In addition, if larvae live in habitats that are too polluted, temporary or inaccessible, predation may not be important. Therefore, the geographic patterns observed in this analysis, mainly in male populations, may be determined by local factors, such as food availability and proximity to water bodies.

Several studies of culicids have shown that wing shape is an indicator of microevolution. Although most quantitative genetic information cannot be obtained from wings (Dujardin 2008), morphometric analyses of wing size and shape are suitable and can serve as a preliminary assessment of evolutionary patterns in culicids (Lorenz et al. 2017). Two and 4 yr were sufficient for *Ae. albopictus* and *Ae. aegypti*, respectively, to accumulate wing shape changes (Vidal et al. 2012, Louise et al. 2015). Therefore, the 100 yr that *Cx. pipiens* s.s. have been on Rapa Nui may be sufficient to indicate microevolutionary processes as a result of its extreme isolation.

The differences found using geometric morphometrics tools between biotypes and geographical areas in *Cx. pipiens* s.s. provide new evidence of the effects of geographic and environmental processes on mosquitoes of health importance.
